# Kernel machine tests of association between brain networks and phenotypes

**DOI:** 10.1371/journal.pone.0199340

**Published:** 2019-03-21

**Authors:** Alexandria M. Jensen, Jason R. Tregellas, Brianne Sutton, Fuyong Xing, Debashis Ghosh

**Affiliations:** 1 Department of Biostatistics & Informatics, University of Colorado Anschutz Medical Campus, Aurora, Colorado, United States of America; 2 Department of Psychiatry, University of Colorado Anschutz Medical Campus, Aurora, Colorado, United States of America; 3 Research Services, Denver VA Medical Center, Aurora, Colorado, United States of America; 4 Department of Behavioral Health, Denver Health, Denver, Colorado, United States of America; University of California San Francisco, UNITED STATES

## Abstract

Applications of quantitative network analysis to functional brain connectivity have become popular in the last decade due to their ability to describe the general topological principles of brain networks. However, many issues arise when standard statistical analysis techniques are applied to functional magnetic resonance imaging (fMRI) connectivity maps. Frequently, summary measures of these maps, such as global efficiency and clustering coefficients, collapse the changing structures of graph topology from many scales to one. This can result in a loss of whole-brain spatio-temporal pattern information that may be significant in association and prediction analyses. Drawing from the electrical engineering field, the resistance perturbation distance is a quantification of similarity between graphs on the same vertex set that has been shown to identify changes in dynamic graphs, such as those from fMRI, while not being computationally expensive or result in a loss of information. This work proposes a novel kernel-based regression scheme that incorporates the resistance perturbation distance to better understand the association with biological phenotypes from fMRI using both simulated and real datasets.

## Introduction

Since its introduction in the early 1990s [1] [2] [3], functional magnetic resonance imaging (fMRI) has rapidly grown to become the most popular technique to observe the living human brain [4]. Noninvasive, in-vivo techniques like fMRI can be used in biomedical research to examine localization of brain regions engaged by a particular task, determining brain networks, and predicting psychological or disease states [5]. While most fMRI studies initially focused on the examination of brain regions engaged during a specific task, increased attention has been paid in examining the connectivity of the entire brain at rest, commonly referred to as resting state fMRI (rs-fMRI) [6]. Analysis of rs-fMRI can help yield information about the strength of connections within and among brain regions that may be unique to clinical populations [6] [7]. All of these objectives can be achieved through the application of statistical techniques that address the specific complications that arise from analysis of spatially correlated, four-dimensional data.

The most common technique used to analyze fMRI data is a mass univariate analysis (MUA). In MUA, a general linear model is fit at each voxel independently with a combination of experimental conditions and biological confounders as covariates [8]. This creates a map of parameter estimates and test statistics that is then thresholded to identify significant voxels; the location and clustering of these significant voxels inform the functional relationships within the brain. Time series methods have also been successfully implemented to examine the interregional correlation values over the length of the fMRI scan [6]. However, these techniques generally ignore the underlying spatial relationships within the data; even though voxel responses are correlated, mass univariate analysis and its time series extension do not fully account for the underlying spatial correlation [8]. However, by extending the general linear model framework to allow for the modeling of non-linear relationships, more complex associations can be fit. One such way to accomplish this is through the use of kernels, which are weighting functions used to estimate the conditional expectation of a random variable [9].

In contrast to the use of general linear models and kernel regression, applications of quantitative network analysis through graph theory have become popular in the last decade due to their utility in describing the general topological principles of brain networks [10]. The application of graph theory to study the underlying structural and functional connections within the brain was first introduced by Ed Bullmore and Olaf Sporns in their seminal work, published in 2009 [10]. In their work, Bullmore and Sporns showed how connectivity analyses can be used not only to analyze structural networks that represent the architectural connections within and between regions, but also to analyze the underlying functional networks that can elucidate how this architecture supports various neurophysiological dynamics [10]. Numerous studies have reported that brain network parameters, derived from fMRI, EEG/MEG, and structural MRI, differ between subjects based on task or underlying biological or physiological condition [10]. However, many issues arise when applying standard statistical methods to fMRI connectivity maps. Frequently, summary measures of these maps, such as global efficiency and clustering coefficients, collapse the changing structure of graph topology from many scales to one [11]. This can result in a loss of whole brain spatiotemporal pattern information that may be significant in association and prediction analyses.

This study proposes a kernel regression scheme that incorporates the resistance perturbation distance to better predict biological phenotypes from fMRI using both simulated and real datasets. Drawing from the electrical engineering field, the resistance perturbation distance (RPD) is a quantification of similarity between graphs on the same vertex set that has been shown to identify changes in dynamic graphs, such as those from fMRI, while not being computationally expensive or result in a loss of information [11]. By incorporating the RPD into a kernel distance function, the high-dimensional feature space of brain networks, defined on input pairs, can be generalized to non-linear spaces; this allows for a wider class of distance-based algorithms, rather than the restrictive squared distance, to be representative of the similarity between two networks. We hypothesize that this algorithm will show significant associations between the metric and phenotype.

The remainder of this paper is organized as follows. The methods section will describe the derivation and properties of the resistance perturbation distance, the general framework for distance-based kernels, and a kernel-based score test. We then apply these methods under a variety of simulation paradigms and to the COBRE-I dataset. Finally, we conclude the paper with a discussion and proposal of future directions. All R code has been made available as supplementary material to this manuscript in the form of a Github repository.

## Materials and methods

### The resistance perturbation distance

Like many complex systems, the human brain is highly dynamic, where the relationship between regions changes with respect to time. The brain is a highly plastic organ, able to reorganize itself through modifications of its neuronal connections. This feature is unique to the central nervous system: neuroplasticity occurs at the beginning of life, where the immature brain organizes itself, when it is subjected to trauma or injury, and throughout adulthood whenever something new is learned. The most popular graph theory measures collapse this complex system from many scales to one, resulting in a loss of information. In contrast, the resistance perturbation distance is a quantification that is flexible enough to account for configurational changes that can occur on a local scale (through local neighbors on the same node) or on a global scale (through connections between clusters or hubs) [11].

Let *G* = (*V*, *E*, *w*), be an undirected, weighted graph that is connected and contains no self-loops, where *V* = {1, 2, …, *n*} is the vertex set, *E* is the edge set, and *w* is a symmetric weight function that provides a quantification of the strength of the relationship between two vertices. The higher the value of *w*, the stronger the relationship between two vertices *i* and *j*. If we define the weighted adjacency matrix to be

$$\begin{array}{c} A_{ij}=A_{ji}=\{\begin{array}{c} w_{e}\;\text{if}\;\text{the}\;\text{edge}\;e=[i,j]\in E\\ 0\;\text{otherwise}, \end{array} \end{array}$$ (1)

the degree matrix’s definition is simply $D_{ii}=\sum_{j=1}^{n}A_{ij}$ [11]. Using the adjacency and degree matrices, the Laplacian matrix, **L**, is a symmetric and positive semi-definite matrix, where **L** = **D** − **A** [11]. If the Moore-Penrose pseudo-inverse of **L**, denoted **L**^**†**^, is found, then, by definition of **L**, **L**^**†**^ is also symmetric.

An important aspect to these adjacency matrices is that a family of distances on *G*^(1)^ and *G*^(2)^ can be induced through the application of a matrix-to-matrix function *ϕ* on the corresponding adjacency matrices. Monnig and Meyer define a general graph distance as

“Given a matrix-to-matrix function, *φ*, on a square matrix, *M*_*n*×*n*_,

$$\begin{array}{c} \varphi =M_{n\times n}\to M_{n\times n} \end{array}$$

and a distance *d* on *M*_*n*×*n*_, we define the pseudo-distance *d*_*φ*_ between two graphs *G*^(1)^ and *G*^(2)^ as

$$\begin{array}{c} d_{\varphi }(G^{\left(1\right)},G^{\left(2\right)})=d[\varphi \left(A^{\left(1\right)}\right),\varphi \left(A^{\left(2\right)}\right)] \end{array}$$

where *A*^(1)^ and *A*^(2)^ are the adjacency matrices of *G*^(1)^ and *G*^(2)^, respectively. If *φ* is injective, then *d*_*φ*_ defines a distance [11].”

This definition is important in that it decouples two important aspects to the distance *d*_*ϕ*_. The matrix-to-matrix function *ϕ* extracts geometric or configurational properties from each graph while the distance *d* can be used to emphasize the relative size of variations in *ϕ*. Koutra et al. expand on this definition, defining axioms that any distance measure should satisfy:

1. *d*_*φ*_(*G*^(1)^, *G*^(1)^) = 0
2. *d*_*φ*_(*G*^(1)^, *G*^(2)^) = *d*_*φ*_(*G*^(2)^, *G*^(1)^)
3. *d*_*φ*_(*G*^(1)^, *G*^(2)^) → 0 as the number of nodes *v* → ∞, where the edge sets between *G*^(1)^ and *G*^(2)^ are complementary [12].

As well, a distance measure should satisfy the following properties:

1. Edge Importance: a change in an edge that creates disconnected components within the graph should be penalized more than changes that maintain its connectivity properties.
2. Weight Awareness: the larger the weight of a removed edge, the greater its impact on the distance.
3. Edge-“Submodularity”: a change is more meaningful in a sparse graph than in a denser graph that are both defined on the same vertex set.
4. Focus Awareness: random changes in a graph result in a smaller impact than targeted changes [11] [12].

The concept of effective resistance, commonly seen in the electrical engineering field, can be extended to the graph theoretic measure of path length but with a richer choice of distance *d*. Monnig and Meyer showed that the effective resistance between two vertices falls under the definition of a general graph distance, but also preserves the three axioms and four properties detailed above. Because the BOLD signal measures the indirect correlate of neuronal responses in the brain, seeing the brain as a circuit board is not an uncommon analogy. Therefore, the use of effective resistance can be easily extended to the summarization of fMRI data. Effective resistance can be defined as

$$\begin{array}{c} \begin{array}{cc} & R=diag(L^{†})1^{T}+1diag{\left(L^{†}\right)}^{T}-2L^{†},\\ & diag(L^{†})=[\begin{array}{c} L_{11}^{†}\\ L_{22}^{†}\\ \vdots \\ L_{nn}^{†} \end{array}] \end{array} \end{array}$$ (2)

[11].

Using this, the resistance perturbation distance is then defined as the element-wise p-norm of the difference between effective resistances such that

$$\begin{array}{c} d_{rp(p)}(G^{\left(1\right)},G^{\left(2\right)})={∥R^{\left(1\right)}-R^{\left(2\right)}∥}_{p}={\left[\sum_{i,j\in V}{\left|R_{ij}^{\left(1\right)}-R_{ij}^{\left(2\right)}\right|}^{p}\right]}^{\frac{1}{p}} \end{array}$$ (3)

for 1 ≤ *p* < ∞ [11].”

This metric defines a distance on the space of connected, undirected, weighted graphs on the same vertex set, where **R** is fully and uniquely defined by **L** and the element-wise p-norm, ‖⋅‖_*p*_, is a norm for *M*_*n*×*n*_. Thus, the distance can easily be shown to be non-negative and symmetric by application of the definition of an element-wise p-norm and satisfies the triangle inequality,

$$\begin{array}{c} {∥R^{\left(1\right)}-R^{\left(2\right)}∥}_{p}\leq {∥R^{\left(1\right)}∥}_{p}-{∥R^{\left(2\right)}∥}_{p}. \end{array}$$ (4)

Additionally, if we observe *G*^(1)^ = *G*^(2)^, then *d*_*rp*(*p*)_(*G*^(1)^, *G*^(2)^) = 0 because **R**^(1)^ = **R**^(2)^ by definition. Conversely, if *G*^(1)^ and *G*^(2)^ are two graphs with the same effective resistance matrix, **R**^(1)^ = **R**^(2)^, this implies the equality of the Laplacian matrices, **L**^(1)^ = **L**^(2)^ and, continuing on this train of thought, equality of their weighted adjacency matrices, **A**^(1)^ = **A**^(2)^ [11].

### Distance-based kernels and a kernel-based score test

Rather than assume a parametric form in the relationship between functional connectivity matrices and phenotypic classification, kernel distance estimation is a non-parametric way to quantify the similarity between data instances. A range of kernel functions are used in statistics, where the choice of kernel determines the function space used to approximate the relationship between two variables [13]. A distance-based kernel is denoted as

$$\begin{array}{c} K_{d}(x_{1},x_{2})=exp\{\frac{-d^{2}\left(x_{1},x_{2}\right)}{\rho }\}, \end{array}$$ (5)

where *d*^2^(*x*_1_, *x*_2_) is a distance function and *ρ* an unknown bandwidth or scaling parameter [9]. *K*_*d*_(*x*_1_, *x*_2_) can be thought of as a measure of similarity between two subjects *x*_1_, *x*_2_ in terms of some common, underlying multidimensional variable set *Z*. This similarity measure can then be incorporated into a regression framework to test to what extent variation in *Z* can explain variation in the outcome, *Y*. An important underlying assumption of this framework, however, is that the distance metric is positive, symmetric, and semi-definite. Unlike other commonly used distance metrics, like the edit distance [14], the RPD satisfies this property and, therefore, can be utilized within a kernel function.

In the case of a dichotomous outcome, assume a logistic regression framework of the semiparametric form

$$\begin{array}{c} logit[Pr(Y_{i}=1)]=X_{i}^{T}\beta_{i}+k(Z_{i})+\epsilon_{i}, \end{array}$$ (6)

where *X*_*i*_ is a matrix of covariates whose association to the dichotomous outcome, *Y*_*i*_, is to be parametrically estimated, *k*(⋅) is a centered, smooth kernel function, and *Z*_*i*_ is a vectorized form of the RPD matrix from the previous section [13] [9]. An important feature of *k*(*Z*_*i*_) is that it lies within a Reproducing Hilbert Kernel Space (RHKS). A hypothesis test can be conducted to determine whether the multidimensional variable set *Z*_*i*_ is associated with *Y*, controlling for *X*, of the form

$$\begin{array}{cc} & H_{0}:k(\cdot )=0\\ & H_{A}:k(\cdot )\neq 0 \end{array}$$

[13] [9].

Assuming that *k*(⋅) lies within a RHKS, $k(\cdot )\in H_{k}$, *β* and *k*(⋅) can be simultaneously estimated by maximizing the penalized log likelihood function

$$\begin{array}{c} \begin{array}{cc} \ell [\beta ,k(\cdot )] & =\sum_{i=1}^{n}[y_{i}log(\frac{\mu_{i}}{1-\mu_{i}})+log(1-\mu_{i})]-\frac{\lambda }{2}{∥k∥}_{H_{k}}^{2}\\ & =\sum_{i=1}^{n}[Y_{i}(X_{i}^{T}\beta_{i}+k(Z_{i}))-log(1+exp\{X_{i}^{T}\beta_{i}+k(Z_{i})\})]-\frac{\lambda }{2}{∥k∥}_{H_{k}}^{2}, \end{array} \end{array}$$ (7)

where λ is a regularization parameter that reflects the trade off between model complexity and goodness of fit [15]. At its boundaries, λ = 0 reflects a saturated model, while λ = ∞ reduces the model to a fully parametric logistic regression model. However, it should be noted that there are two unknown parameters within *ℓ*[*β*, *k*(⋅)]: the regularization parameter λ and bandwidth parameter *ρ*. Intuitively, λ controls the magnitude of the unknown function *k*(⋅) while *ρ* controls the smoothness of *k*(⋅) [13]. The choice of *ρ* has a strong influence on the resulting estimate and choosing the data-driven, minimally optimal value of *ρ* is crucial. Using the representer theorem, which states that a solution to the penalized log likelihood function

$$\begin{array}{c} \min_{k(\cdot )\in H_{k}}[\ell_{y}(k(x_{1}),...,k(x_{n}))+\Omega {∥k∥}_{H_{k}}^{2}] \end{array}$$ (8)

takes the form

$$\begin{array}{c} k^{*}(Z_{i})=\sum_{j=1}^{n}\alpha_{j}K(x_{i},x_{j})=K_{i}^{T}\alpha , \end{array}$$ (9)

[16] then the penalized log likelihood function can be rewritten as

$$\begin{array}{c} \ell [\beta ,k(\cdot )]=\sum_{i=1}^{n}[Y_{i}(X_{i}^{T}\beta_{i}+k(Z_{i}))-log(1+exp\{X_{i}^{T}\beta_{i}+k(Z_{i})\})]-\frac{\lambda }{2}\alpha^{T}K\alpha . \end{array}$$ (10)

Solving for *α* and *β* gives the closed form equations

$$\begin{array}{c} \begin{array}{cc} & \hat{\alpha }=\frac{1}{\lambda }{\left(I+\frac{K}{\lambda }\right)}^{-1}(Y-X\hat{\beta })\\ & \hat{\beta }={\left[X^{T}{\left(I+\frac{K}{\lambda }\right)}^{-1}X\right]}^{-1}X^{T}{\left(I+\frac{K}{\lambda }\right)}^{-1}Y \end{array} \end{array}$$ (11)

and then, plugging in $\hat{\alpha }$ into k*(*Z*_*i*_),

$$\begin{array}{c} {\hat{k}}^{*}\left(Z\right)=\frac{1}{\lambda }\{K\left(Z,Z_{1}\right),\cdots ,K\left(Z,Z_{n}\right)\}{\left(I+\frac{K}{\lambda }\right)}^{-1}(Y-X\hat{\beta }) \end{array}$$ (12)

[13]. However, it is possible to approach *ℓ*[*β*, *k*(⋅)] from a generalized linear mixed models (GzLMM) perspective. As logistic regression is a special case of GzLMM, the kernel estimator within the semiparametric logistic regression model parallels the penalized quasi-likelihood function from a logistic mixed model, letting *τ* = 1/λ denote the regularization parameter and *ρ* remaining the bandwidth parameter [13]. These parameters can be treated as variance components, where *k*(⋅) ∼ *N*(0, *τK*(*ρ*)) can be treated as a subject-specific random effect and the covariance matrix *K*(*ρ*) is an *n* × *n* kernel matrix as previously defined [15]. This then means that estimating *β* and *k*(⋅) can be done by maximizing the penalized log likelihood

$$\begin{array}{c} \ell [\beta ,k(\cdot )]=\sum_{i=1}^{n}[Y_{i}(X_{i}^{T}\beta_{i}+k(Z_{i}))-log(1+exp\{X_{i}^{T}\beta_{i}+k(Z_{i})\})]-\frac{1}{2\tau }h^{T}Kh, \end{array}$$ (13)

where *h* = *Kα* and *τ* = 1/λ [15]. This provides an advantage as it allows for testing of the null hypothesis *H*_0_: *τ* = 1/λ = 0 without explicit specification of basis functions.

However, under the null hypothesis, the kernel matrix *K* disappears, which makes *ρ* a nuisance parameter that is inestimable under the null hypothesis. Davies studied the issue of a nuisance parameter disappearing under the null hypothesis [17], and proposed a score test be used. The score statistic is treated like a nuisance parameter-indexed Gaussian process. As Eq 10 is a nonlinear function of (*α*, *β*), a Newton-Raphson algorithm needs to be implemented to maximize Eq 10 in terms of (*α*, *β*). If (*j*) is the *j*^*th*^ iteration of the algorithm, then the (*j* + 1) step solves

$$\begin{array}{c} \left[\begin{array}{cc} X^{T}D^{\left(j\right)}X & X^{T}D^{\left(j\right)}K\\ D^{\left(j\right)}X & \tau^{-1}I+D^{\left(j\right)}K \end{array}][\begin{array}{c} \beta^{\left(j+1\right)}\\ \alpha^{\left(j+1\right)} \end{array}]=[\begin{array}{c} X^{T}D^{\left(j\right)}{\tilde{y}}^{\left(j\right)}\\ D^{\left(j\right)}{\tilde{y}}^{\left(j\right)} \end{array}\right] \end{array}$$ (14)

where ${\tilde{y}}^{\left(j\right)}=X\beta^{\left(j\right)}+K\alpha^{\left(j\right)}+{\left(D^{\left(j\right)}\right)}^{-1}(y-X_{i}^{T}\beta^{\left(j\right)})$, $D^{\left(j\right)}=diag[X_{i}^{T}\beta^{\left(j\right)}-(1-X_{i}^{T}\beta^{\left(j\right)})]$, and *h*^(*j*)^ = *Kα*^(*j*)^. Also noting that *β* and *k*(⋅), which depend on *τ* and *ρ*, can be estimated using penalized quasi-likelihood under a logistic mixed model paradigm, then rewriting Eq 13

$$\begin{array}{c} \ell (\beta (\nu ),\nu )\approx -\frac{1}{2}log\left|V\right|-\frac{1}{2}log\left|X^{T}V^{-1}X\right|-\frac{1}{2}{\left(\tilde{y}-X\beta \right)}^{T}V^{-1}(\tilde{y}-X\beta ), \end{array}$$ (15)

where *ν* = (*τ*, *ρ*) and *V* = *D*^−1^ + *τK*. Then $\hat{\nu }$ can be solved for in the usual way [15]. However, if the derivative of (9) is taken with respect to *τ*, then the score test for *H*_0_: *τ* = 1/λ = 0 can be written as

$$\begin{array}{c} S(\rho )=\frac{Q_{\tau }(\hat{\beta_{0}},\rho )-\mu_{Q}}{\sigma_{Q}} \end{array}$$ (16)

where $Q_{\tau }(\hat{\beta_{0}},\rho )={\left(\tilde{y}-X\hat{\beta_{0}}\right)}^{T}DK\left(\rho \right)D(\tilde{y}-X\hat{\beta_{0}})={\left(\tilde{y}-\mu_{0}\right)}^{T}K(\tilde{y}-\mu_{0})$, $\hat{\beta_{0}}$ is the maximum likelihood estimate of *β* under the null hypothesis, $\hat{\mu_{0}}=logit^{-1}(X\hat{\beta_{0}})$, *μ*_*Q*_ = *trace*[*P*_0_*K*(*ρ*)], $\sigma_{Q}^{2}=2trace[P_{0}K\left(\rho \right)P_{0}K\left(\rho \right)]$, *P*_0_ = *D*_0_ − *D*_0_*X*(*X*^*T*^ *D*_0_*X*)^−1^ *X*^*T*^ *D*_0_, and *D*_0_ = *diag*[*μ*_0_ − (1 − *μ*_0_)] [15].

*S*(*ρ*) under the null hypothesis is an approximate, *ρ*-indexed Gaussian process, which application of Davies’ results [17] to get the upper bound for the score test’s p-value. It can be seen that large values of $Q_{\tau }(\hat{\beta_{0}},\rho )$ would result in a rejection of *H*_0_ and the upper bound of the p-value is

$$\begin{array}{c} \Phi (-M)+\frac{Wexp\{-\frac{1}{2}M^{2}\}}{\sqrt{8\pi }} \end{array}$$ (17)

where Φ(⋅) is the normal cumulative distribution function, *M* is the maximum of *S*(*ρ*) over all of the searched range of *ρ*, *W* = |*S*(*ρ*_1_) − *S*(*L*)| + |*S*(*ρ*_2_) − *S*(*ρ*_1_)| + ⋯ + |*S*(*U*) − *S*(*ρ*_*m*_)|, *L* and *U* are the lower and upper bounds, respectively, of the search area for *ρ*, and *ρ*_*m*_ are the search points between *L* and *U* [17] [15]. Liu et al. suggest setting the lower and upper bounds of the *ρ* search to be $L=0.1min_{i\neq j}\sum_{l=1}^{p}{\left(z_{il}-z_{jl}\right)}^{2}$ and $U=100max_{i\neq j}\sum_{l=1}^{p}{\left(z_{il}-z_{jl}\right)}^{2}$ [15].

## Results

### Simulations

Functional connectivity matrices were simulated using the MNS package in R. This package uses the mixed neighborhood selection (MNS) algorithm, which separates a network into two components: common population and subject-specific edges [18]. Using the preferential attachment model proposed by Barabási and Albert in 1999, a set of edges, denoted *E*^*pop*^, is shared across all subjects in the sample, where edge strengths are uniformly sampled [18]. Inter-subject variability among the edges, denoted $\bar{E}$, are chosen according to the Erdös and Rényi model, where a choice of the number of elements of $\bar{E}$ determines the number of random edges and, thus, the level of inter-subject variability [18].

To simulate the data that corresponds to the functional connectivity networks, a multivariate normal distribution is utilized,

$$\begin{array}{c} X^{\left(i\right)}∼N(0,{\left[PD(\Theta^{pop}+\Theta^{\left(i\right)})\right]}^{-1}), \end{array}$$ (18)

where *PD*(⋅) is a function that ensures a positive definite standard deviation matrix, Θ^*pop*^ denotes the population networks, and Θ^(*i*)^ denotes the subject-specific networks.

**Fig 1:**
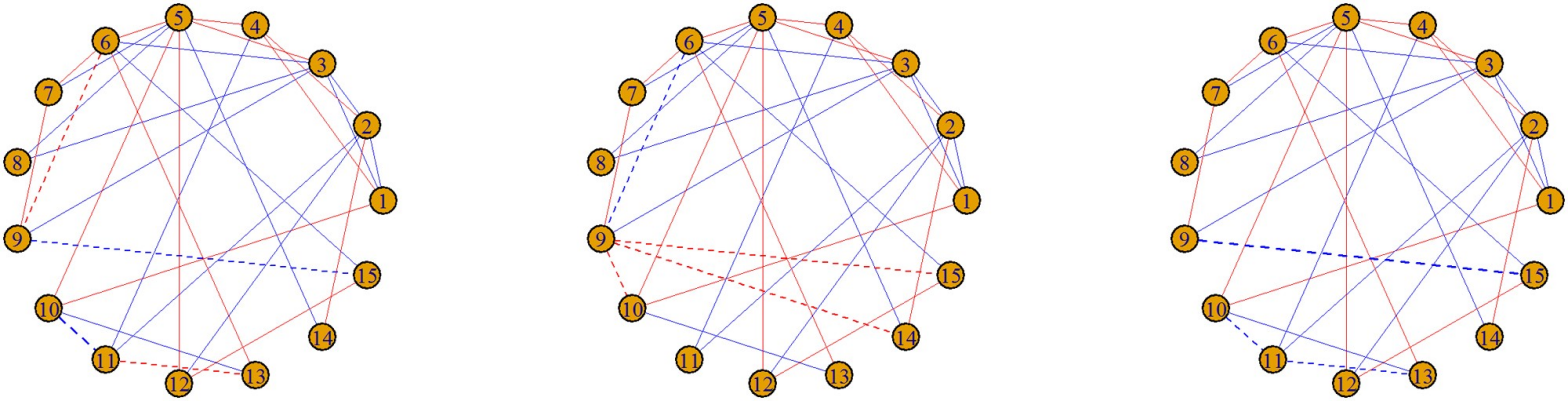
Simulated networks for N = 3 subjects under the “cohort” method. Solid lines between nodes represent population edges and dashed lines represent subject-specific edges. Red edges represent a positive association between edges while blue edges represent negative associations. The following command was used: gen.Network(method = “cohort”,p = 15,Nsub = 3,sparsity = 0.2,REsize = 10, REprob = 0.5,REnoise = 1).

To simulate the functional connectivity data using the MNS package, the gen.Network() function was called; the parameters associated with the gen.Network() are detailed in the MNS package documentation [18]. The result is an S3 object of class MNS. Fig 1 below shows an example of simulated functional connectivity networks for three subjects.

For all simulations, the following settings were utilized in the gen.Network() function, which seem to best represent the variability in functional connectivity networks from resting state MRI datasets: p = 90, sparsity = 0.75, REsize = 10, REprob = 0.65, REnoise = 3.

However, the data simulated in the MNS package does not exactly match data from a resting state fMRI scan. The existence of negative correlations between brain networks has been a hotly contested debate within the neuroimaging community; the origin, interpretation, and link to the underlying structural connectivity are still unresolved issues. Because of this, the norm within the field is to zero out any negative correlations within the connectivity matrices before further analysis is performed. Other options include taking the absolute value or to normalize the correlations to be between 0 and 1, although these are far less popular. For the purposes of this manuscript, simulated datasets from the MNS package had all negative correlations zeroed out.

**Fig 2:**
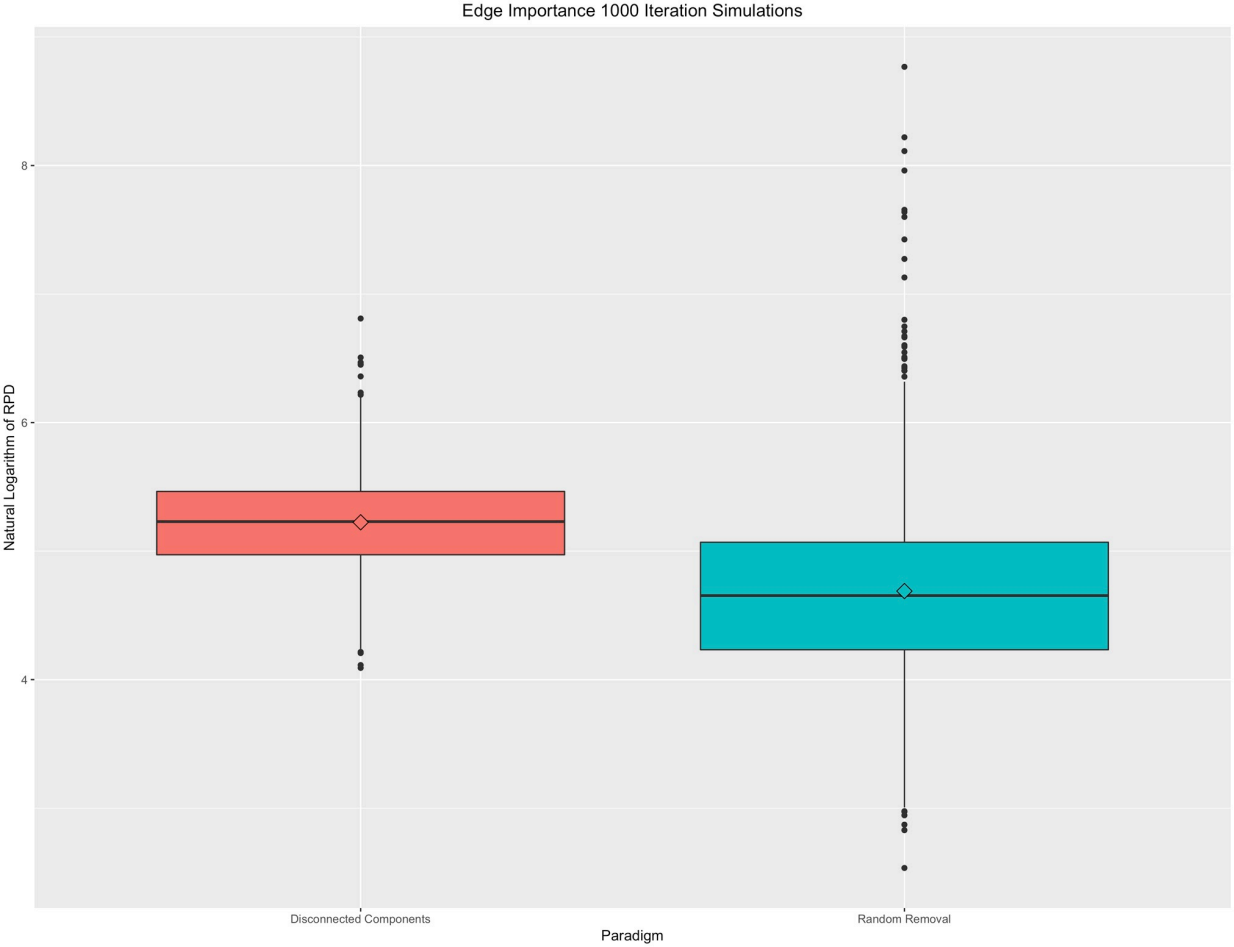
Edge importance property. Boxplot of 1000 iterations under targeted deletions resulting in disconnected components and equally-numbered, but random, deletions. A natural logarithm transformation has been applied to all resistance perturbation distance values for this figure. While random deletions result in a wider overall spread of distances, targeted deletions result, on average, in a larger RPD than a comparable random deletion.

The four properties of a distance—edge importance, weight awareness, edge-“submodularity,” and focus awareness—were tested for the resistance perturbation distance under varying simulation models of ten-node connectivity matrices. Under the edge importance property, in weighted graphs, changes that created disconnected components should be penalized more than changes that maintain the connectivity properties of the graph. We simulated this property by breaking up the simulated connectivity matrix into four, equally-sized quadrants and then zeroing out all non-zero cells within the off-diagonal (quadrants I and III) to create two disconnected components. Then, the same number of components were randomly zeroed out to create a comparable “random” graph. Pairwise RPDs were plotted for 1000 iterations in Fig 2, below.

**Fig 3:**
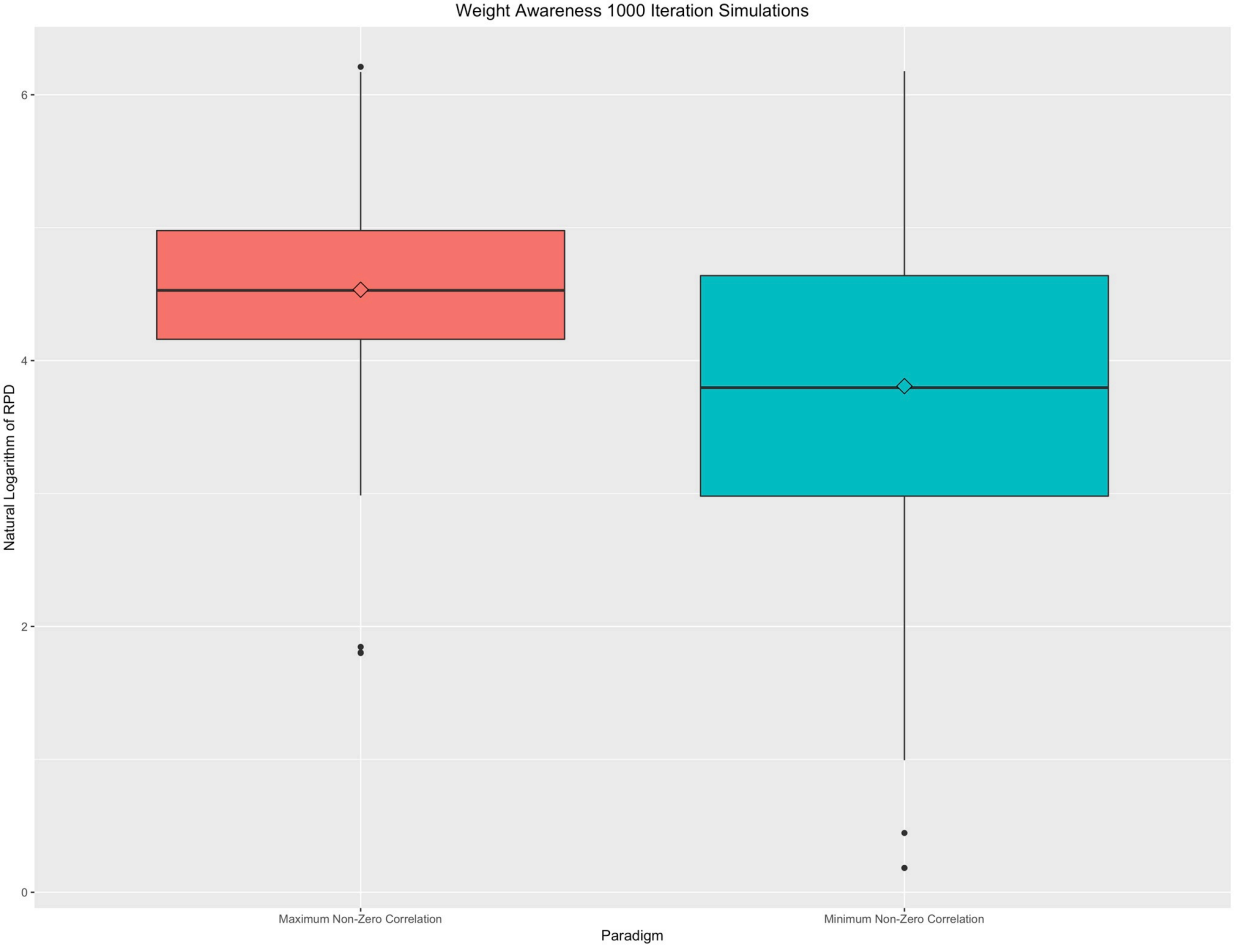
Weight awareness property. Boxplot of 1000 iterations under minimum and maxiumum non-zero correlation paradigms. A natural logarithm transformation has been applied to all resistance perturbation distance values for this figure. While minimum non-zero deletions result on a wider overall spread of distances, the maximum non-zero correlation deletions result, on average, in a larger RPD than a comparable minimum non-zero correlation deletion.

Under the weight awareness property, in weighted graphs, the larger the weight of the removed edge, the greater the impact on the distance. Minimum and amximum non-zer ocorrelations were iteratively zeroed out from a simulated connecitivty matrix. Pairwise RPDs were plotted for 1000 iterations in Fig 3, below.

**Fig 4:**
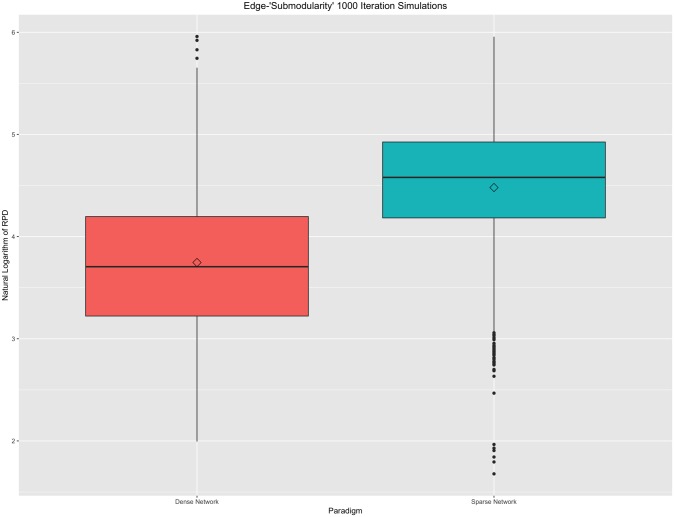
Edge-“submodularity” property. Boxplot of 1000 iterations under sparse and dense graph paradigms. A natural logarithm transformation has been applied to all resistance perturbation distance values for this figure. Random deletions from dense graphs, on average, result in smaller RPDs than in comparable sparse graphs.

Under the edge-“submodularity” property, in weighted graphs, a specific change is more important in a graph with few edges than in a much denser, but equally sized, graph. The maximum non-zero correlation was systematically removed from each iteratively-simulated connectivity matrix. Within the gen.Network() function, sparsity parameters of 0.45 (for a sparse graph) and 0.95 (for a dense graph) were set. Pairwise RPDs were plotted for 1000 iterations in Fig 4, below.

**Fig 5:**
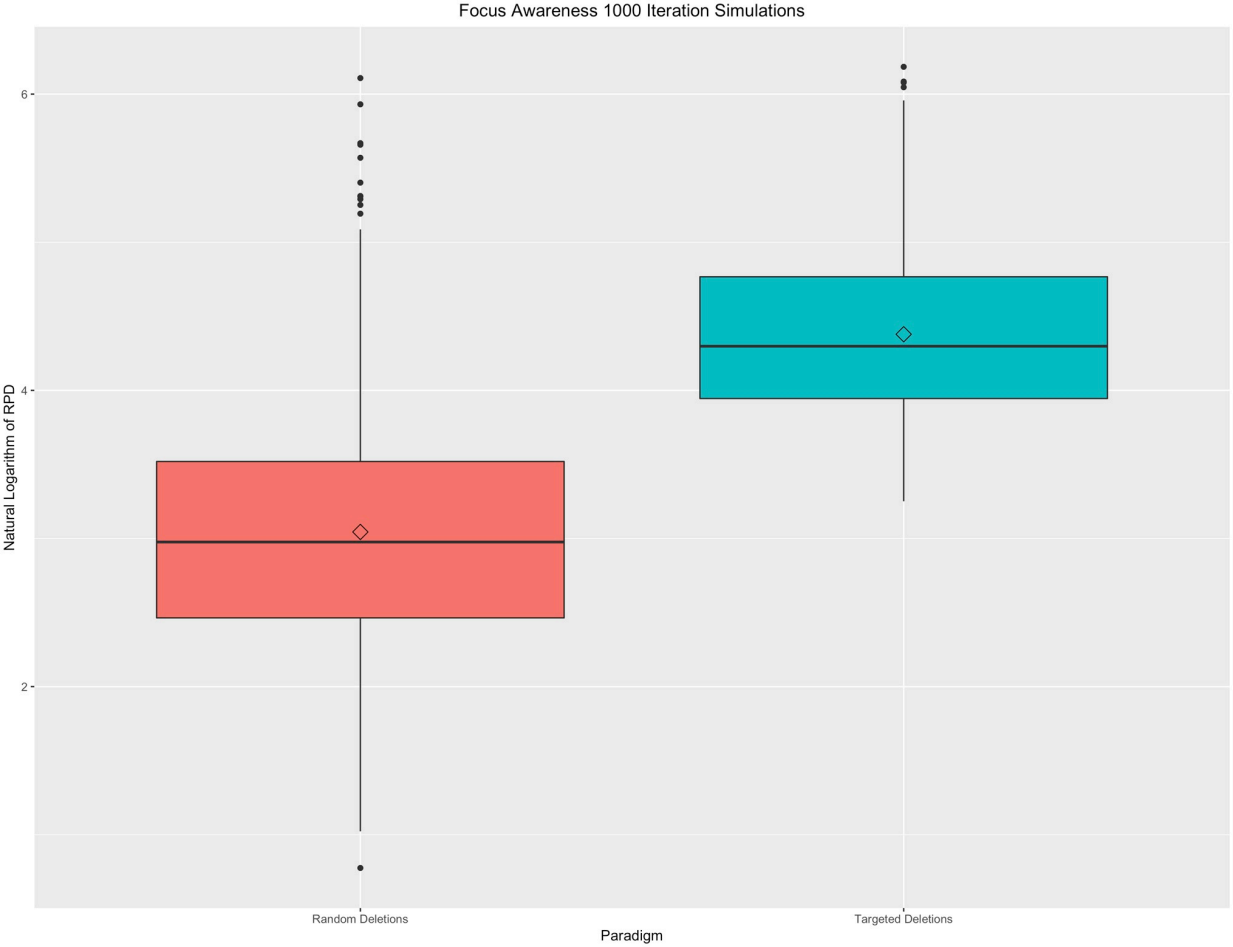
Focus awareness property. Boxplot of 1000 iterations under targeted and random change paradigms. A natural logarithm transformation has been applied to all resistance perturbation distance values for this figure. Targeted deletions, on average, results in larger RPDs than in comparable random graph deletions.

Finally, under the focus awareness property, in weighted graphs, random changes in graphs are less important than targeted changes of the same extent. Similar to Koutra et al. [12], targeted changes were made by deleting all edges from a randomly chosen node while random changes were made by randomly removing the same number of edges from the whole graph. Pairwise RPDs were plotted for 1000 iterations in Fig 5, below. As these figures and tables show, the Koutra et al. properties are all satisfied under the simulated constraints of fMRI data. using the MNS package in R.

Next, to analyze the robustness of the kernel-based score test described, several simulations were conducted. These simulations were split between the number of groups of functional connectivity matrices that were generated; a simulation under a one generation process presumes that the null hypothesis is true (all subjects come from the same underlying population) while a two generation process presumes that the null hypothesis is false and subjects come from two distinct populations. To simplify the analyses for these simulations, no covariates were generated.

A series of simulation studies were conducted to evaluate the performance of the kernel-based score test under the hypothesis test of *H*_0_: *k*(⋅) = 0 versus *H*_*A*_: *k*(⋅) ≠ 0. As there is no closed-form solution for the test statistic’s accompanying p-value, power and Type I error were calculated using simulated datasets. For the power simulation, 100 different datasets were produced, ten from the “control” population, ten from the “patient” population, and the remaining 80 from a third “noise” population. Each of these populations were simulated under different gen.Network() function calls in order to prevent common preferential attachment model parameters. This third population was included to mimic the noisy nature of fMRI data. The noise population was distributed between the “control” and “patient” populations such that the final sample sizes were 55 in the “control” population and 45 in the “patient” population. Each simulated connectivity matrix was generated with the following parameters: p = 90, sparsity = 0.75, REsize = 10, REprob = 0.65, and REnoise = 3. Bounds of the *ρ* search were set based on the suggestion from Liu et al. [15]. An indicator function was used to determine whether each simulation’s resulting p-value was greater than *α* = 0.05. After 1000 iterations, the empirical power of the kernel-based score test was 0.945. Similarly, for the test statistic’s Type I error rate, all 100 simulated samples came from a single generation process with the same parameters as the power simulation and bounds of the *ρ* search. After 1000 iterations, the empirical Type I error rate of the kernel-based score test was 0.0496. These simulations show that the empirically-calculated Type I error is very close to the nominal value of 0.05 while the power of the score test has sufficiently high power to detect true differences in a dataset.

**Fig 6:**
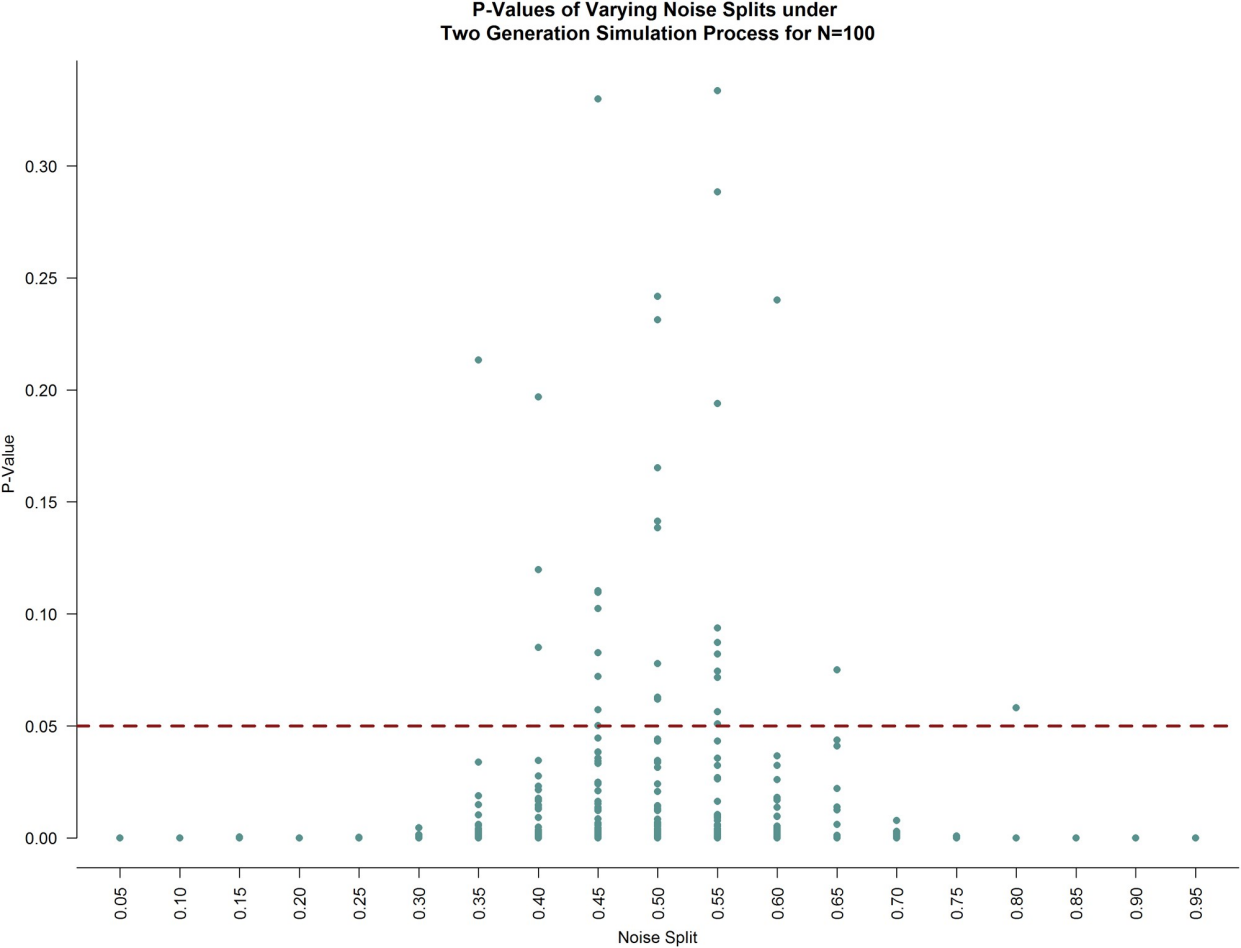
P-values under varying noise population allocations under a two generation simulation process. At each noise allocation, 100 iterations were conducted. The red dashed line is at the nominal p-value of 0.05. Any point above this red line is a false negative while any points below this red line are true positives. The highest proportion of false negatives occurs at noise splits at or surrounding 0.5, as expected.

A simulation was also conducted under a two generation process to understand the impact of how the allocation of the “noise’” population to the “control” and “patient” populations affected the kernel-based score test’s p-values. In a very similar manner to the power simulation, three datasets were produced. However, the allocation of the simulated noise population to the control and patient populations was varied; the percentage of the noise population allocated to the control population varied along (5%, 95%) by increments of 5%. One hundred iterations occurred at each noise allocation. Any iteration that resulted in a p-value greater than 0.05 was considered a false negative as the simulation was set up in such a way that the underlying truth was that the “control” and “patient” populations were different from one another. The results of the simulation are plotted in Fig 6, below. The highest Type II errors occurred between noise splits where 40% and 55% of the noise was allocated to the control population; this is not surprising as a noise allocation that was nearly evenly split between the two groups would make the group average graph look exceedingly similar.

**Fig 7:**
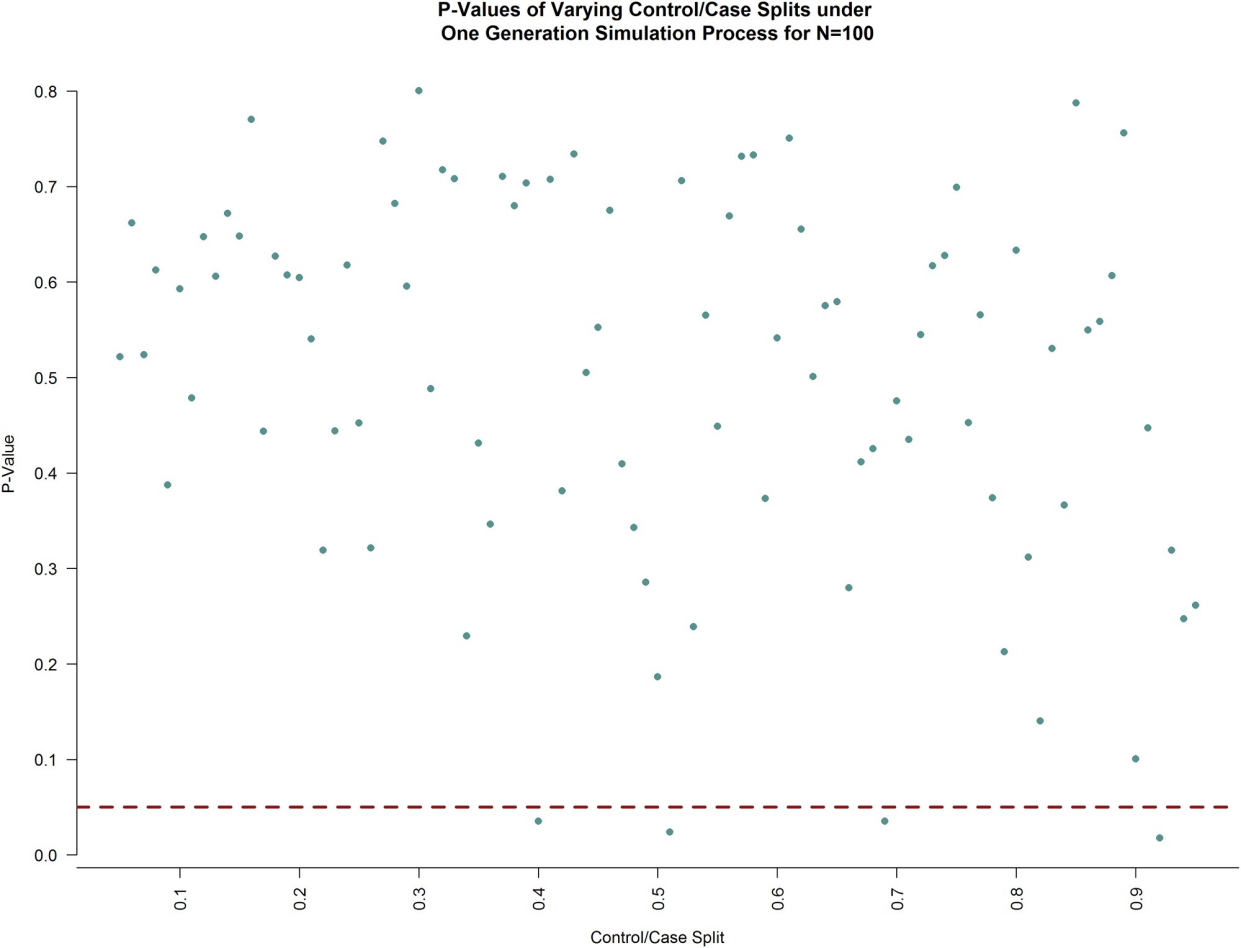
P-values under varying noise population allocations under a one generation simulation process. Allocation of the 100 generated connectivity matrices varied from 5:95 to 95:5, increasing in increments of one. The red dashed line is at the nominal p-value of 0.05. Any point below this red line is a false positive while any point above this red line is a true negative. As can be seen, the false positive rate from this simulation agrees with the Type I error rate previously calculated.

Similarly, a simulation was conducted under a one generation process to understand how splitting the 100 connectivity matrices between “control” and “patient” populations affected the p-values of the kernel-based score test. Like the Type I error simulation, only one dataset was produced. However, how this dataset was split between the two populations was varied; the number of connectivity matrices allocated to the “control” population varied along (5, 95), increasing in increments of one at each iteration. Any iteration that resulted in a p-value less than 0.05 was considered a false positive as the simulation was set up in a way that the underlying truth was that there was no significant difference between “controls” and “patients.” The results of the simulation are plotted in Fig 7, below. The total Type I error across all allocations was 0.05, on par with the empirical Type I error calculated.

### COBRE-I dataset

Created with the focus of studying the neural mechanisms of schizophrenia, the Center for Biomedical Research Excellence through the Mind Research Network for Neurodiagnostic Disvoery (MRN) contributed raw anatomical and functional MR data from 72 patients with diagnosed schizophrenia and 75 healthy controls to the 1000 Functional Connectomes Project [19]. Previous studies [20] [21] of this dataset have shown significant differences between schizophrenia and control patients in the hippocampus and default mode network with more subtle differences in the temporal and frontal networks. However, neither of these studies approached their analysis from a graph theoretic perspective, choosing instead to perform versions of a mass univariate analysis.

Although the initial COBRE-I dataset consisted of 147 subjects, two control patients had to be excluded due to disenrollment [19]. A multi-echo, magnetization prepared rapid gradient echo (MPRAGE) sequence was used to acquire the anatomical information on each subject, with the following parameters: TR/TE/TI = 2530/[1.64, 3.5, 5.36, 7.22, 9.08]/900ms; flip angle = 7°; FOV = 256x256mm; slab thickness = 176mm; matrix = 256x256x176; voxel size = 1x1x1mm; number of echoes = 5; pixel bandwidth = 650Hz, total scan time = 6 minutes [19]. Resting state functional MR data was acquired using a single-shot, full k-space, echo planar imaging (EPI) with ramp sampling correction using the intercommissural line as the reference and the following parameters: TR = 2 seconds; TE = 29ms; matrix size = 64x64; 32 slices; voxel size = 3x3x4mm [19]. In addition to this imaging data, the MRN also provided phenotypic information on each subject, including age, gender, handedness, and diagnostic information, when applicable. Table 1 provides a summary of the phenotypic information on controls and patients.

**Table 1:** COBRE dataset subject demographics.

	Control (n = 73)	Patient (n = 72)
Age in years, mean (SD)	35.90 (11.64)	38.17 (13.89)
Sex, n (%)		
Male	50 (68.5%)	58 (80.56%)
Female	23 (31.5%)	14 (19.44%)
Handedness, n(%)		
Left	1 (1.35%)	10 (13.89%)
Right	71 (95.94%)	60 (83.33%)
Ambidextrous	2 (2.70%)	2 (2.78%)

An automated pre-processing and denoising pipeline was implemented with the CONN software package within MatLab [22]. Within this pipeline, the first four volumes were discarded to ensure T1 equilibrium effects and each subject’s images were realigned to the first volume however no slice-timing correction was applied as images were acquired in a descending manner. Data were spatially normalized to the Montreal Neurological Institute (MNI) space and smoothed using a Gaussian kernel with a full-width at half-maximum of 8mm. During the denoising process, two different sources of possible confounds were regressed out: (1) BOLD signal from white matter and cerebrospinal fluid (CSF); and (2) realignment parameters (6 total). Correlation matrices were then extracted from CONN following a first-level ROI-to-ROI analysis. A hybrid physical atlas was used, where the FSL Harvard-Oxford atlas was used to parcellate the cortical and subcortical areas and the Automated Anatomical Labeling (AAL) atlas [23] was used to to parcellate the cerebellar areas; this resulted in a physical atlas of 132 regions. Weighted networks were extracted from the *.mat files using the R.matlab package. As the matrices contained Fisher’s transformed correlation coefficients, the hyperbolic tangent function was applied to all correlations then negative correlations were set to zero.

Using the entire dataset, which included 72 schizophrenia and and 73 control patients following the pre-processing and denoising procedures, the outcome was a binary classification variable of schizophrenia diagnosis. The regression parameters for the phenotypic covariates of age, sex, and handedness were parametrically estimated while the RPD matrix was non-parametrically estimated. Specifically, we considered the following semiparametric logistic model:

$$\begin{array}{c} logit[Pr(Y=1)]=\beta_{0}+\beta_{1}*age+\beta_{2}*sex+\beta_{3}*handedness+k(RPD) \end{array}$$ (19)

where *k*(⋅) is a nonparametric kernel distance function of the 132 × 132 RPD matrix. Details of the estimation procedure can be found in the Methods section. Additionally, a simpler, fully non-parametric logistic model was considered, which did not include any of the phenotypic covariates:

$$\begin{array}{c} logit[Pr(Y=1)]=k(RPD). \end{array}$$ (20)

This was done to test whether the phenotypic covariates were confounders in the association between the RPD matrix and binary schizophrenia classification.

The same two models (semiparametric and fully non-parametric) were fit to the full dataset, but for which all negative correlations within the subject-level fMRI connectivity matrices left as is. This was done to determine whether there was a significant loss of information by following the neuroimaging standard of zeroing out any negative correlation between regions of interest. Recent articles [24] [25] have pointed to a potentially significant physiological role of negative correlations within fMRI. Specifically, Parente et al. notes that, while these negatively correlated brain networks still lack a well-defined biological explanation, they appear to be have an association with the alterations in brain function in people diagnosed with schizophrenia [24].

The results of these analyses are present in Table 2, below.

**Table 2:** Analysis of full COBRE dataset.

Regression		P-value of score test for *H*_0_: *k*(⋅) = 0
Semiparametric	Zeroing out Negative Correlations	*p* = 0.53
Semiparametric	Keeping Negative Correlations	*p* = 0.61
Fully Non-Parametric	Zeroing out Negative Correlations	*p* = 0.02
Fully Non-Parametric	Keeping Negative Correlations	*p* = 0.48

**Fig 8:**
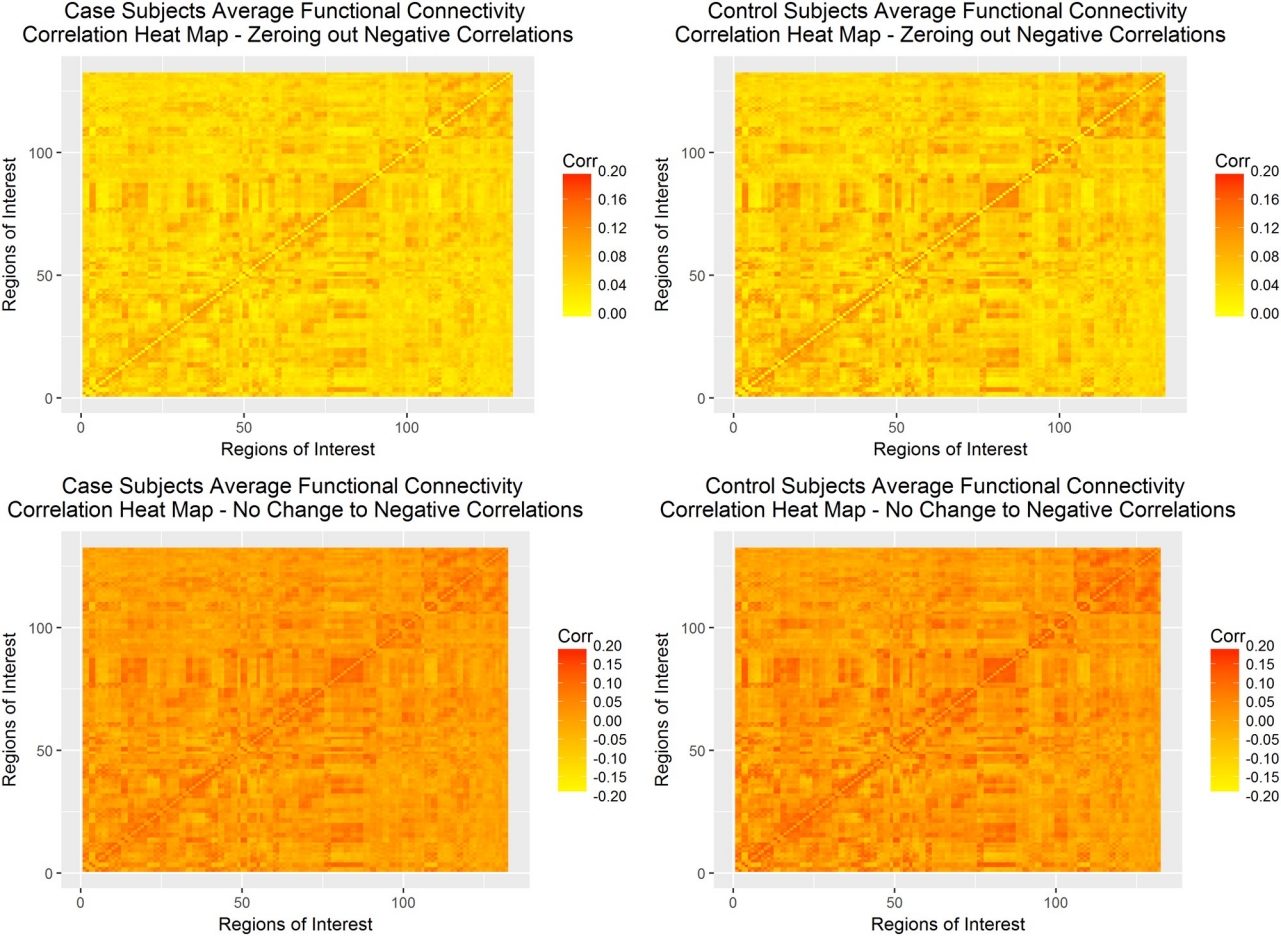
Correlation heat maps full COBRE-I dataset. Correlation heat maps of control and schizophrenia subjects under zeroing out negative correlations (top row) and normalizing all correlations (bottom row).

Table 2 shows that only under the fully non-parametric paradigm when the negative correlations were zeroed out was the null hypothesis of *H*_0_: *k*(⋅) = 0 rejected. The hypothesis that keeping all negative correlations within the dataset would preserve more information was not confirmed as both the semiparametric and fully non-parametric models were not significant at the *α* = 0.05 level. Heat maps of the average connectivity matrix for control versus schizophrenia patients under both paradigms can be seen in Fig 8, below. These heat maps show exceedingly similar patterns of average connectivity between the two groups, which may be the reason why the RPD-based score test was unable to find a significant difference in most of the scenarios we considered.

A subset of the entire COBRE dataset, which included all schizophrenia subjects who had an diagnosis of paranoid schizophrenia (ICD-9 code of 295.3) and an equal number of randomly selected control subjects, was analyzed. To ensure comparable groups, frequency matching for handedness, sex, and age category (18 – 25, 26 – 35, 36 – 45, 46+) was conducted. Because schizophrenia is such a heterogeneous condition, by restricting the sample of cases to only those with the same sub-diagnosis, some of the noise present within the dataset exogenous to the normal variation in fMRI connectivity would be removed. As with the full dataset, four different regression models were fit to the data, the results of which are summarized in Table 3, below.

**Table 3:** Analysis of COBRE dataset—Paranoid schizophrenia cases only.

Regression		P-value of score test for *H*_0_: *k*(⋅) = 0
Semiparametric	Zeroing out Negative Correlations	*p* = 0.49
Semiparametric	Keeping Negative Correlations	*p* = 0.59
Fully Non-Parametric	Zeroing out Negative Correlations	*p* = 0.15
Fully Non-Parametric	Keeping Negative Correlations	*p* = 0.46

**Fig 9:**
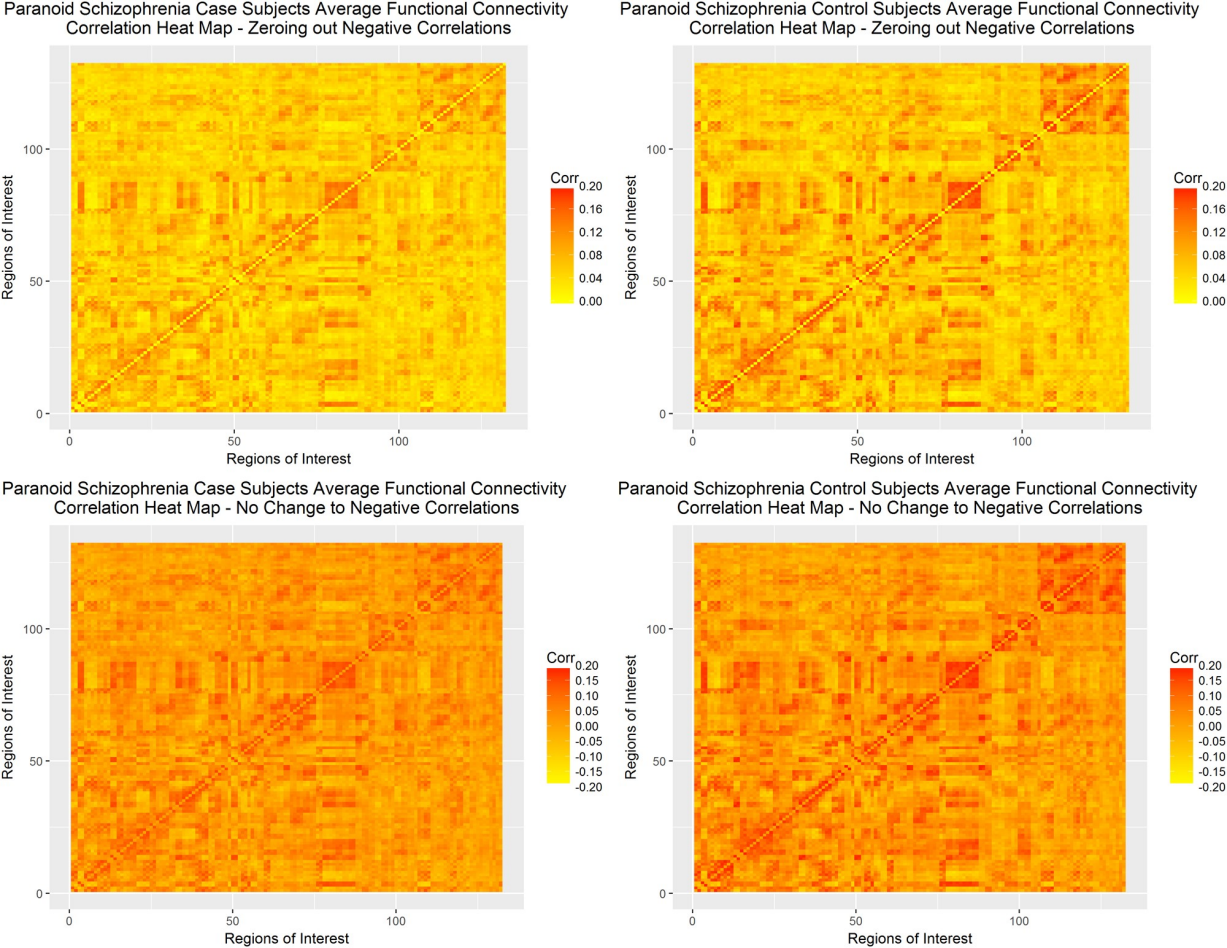
Correlation heat maps paranoid schizophrenia COBRE-I dataset. Correlation heat maps of control and schizophrenia subjects for only the paranoid schizophrenia subset under zeroing out negative correlations (top row) and normalizing all correlations (bottom row).

Table 3 shows that for all four conditions, the null hypothesis of *H*_0_: *k*(⋅) = 0 fails to be rejected. Heat maps of the average connectivity matrix for the randomly-selected control versus paranoid schizophrenia patients under both paradigms can be seen in Fig 9, below. As with the full COBRE dataset, the heat maps do not show gross differences in the totality of functional connectivity. However, in both cases, the schizophrenia patients appear to have lower correlations between regions of interest than the control patients.

As a comparison, the global efficiency and rich club coefficient were calculated for each COBRE-I subject’s connectivity matrix and then included in respective conventional logisitic regression frameworks, where group was the outcome of interest and age, sex, and handedness were included as additional covariates of interest. These regressions were performed for both the full COBRE-I dataset and for the subset of paranoid schoziphrenia subjects. However, because of the way in which these graph theoretic measures are calculated, negative correlations could not remain within the connectivity matrices and, thus, only the condition in which negative correlations were zeroed out couldbe tested. Table 4, shows the results of these regressions for the graph theoretic measure covariate only to provide direct comparison to the kernel logistic regression framework. In all cases, the graph theoretic measure, whether global efficiency or rich club coefficient, were not significantly associated with group label. This lack of significance in these comparison models provides further evidence of the complex nature of the functional connectivity data and how subtle the differences between control subjects and subjects with schizophrenia are.

**Table 4:** Analysis of COBRE dataset—Global efficiency and rich clubs.

Dataset	Graph Theoretic Measure	P-value of Wald Test
Full COBRE-I	Global Efficiency	*p* = 0.06
Full COBRE-I	Rich Club Coefficient	*p* = 0.35
Paranoid Schizophrenia Only	Global Efficiency	*p* = 0.84
Paranoid Schizophrenia Only	Rich Club Coefficient	*p* = 0.99

## Conclusion

### Discussion

In this paper, we applied a concept from the electrical engineering field, the resistance perturbation distance, to a kernel logistic regression framework, where the outcome of interest is a binary classifier, phenotypic covariates are modeled parametrically, and the distance metric is modeled nonparametrically using a kernel machine method. The RPD is computationally efficient and does not result in a loss of information on either a local or global scale, unlike many other graph theoretic measures. The application of a kernel logistic regression allows for the RPD to be modeled without making any assumption as to the parametric form of its association with the binary classifier. Because our model is semi-parametric, we were able to control for potential phenotypic confounders within a parametric framework, allowing for ease of parameter estimate interpretation should they be desired. Further, the kernel regression framework could be extended to account for repeated measures, allowing for RPD metrics to be calculated at multiple points during each subject’s fMRI scan time.

### Limitations and future directions

There are several limitations that affect our approach. First, while our model proved to have high power, a low Type I error rate, and robust to varying study design and searchable spaces of the score test statistics, only one significant association was found between the RPD matrix and the binary classifier in the full COBRE-I dataset under the fully non-parametric score test with negative correlations zeroed out. However, when accounting for multiple comparisons, this association is no longer significant. The difference between simulation and real datasets could be due to a variety of factors, either working in isolation or compounded on one another. Several recently-published studies [7] [26] [27] have noted that choice of pre-processing pipeline can impact the results of an inferential analysis involving graph theoretic measures, especially in resting state fMRI. We have not studied the impact of different parameters within the same pre-processing pipeline nor the impact of an entirely different manner of pre-processing on the RPD. As well, it was noted earlier that, while the overall patterns of connectivity within the heat maps appear to be similar between cases and controls, the overall magnitude of the correlations may differ; the RPD is not sensitive to a global difference in the magnitude of edge weights as it is scale invariant. Finally, while no self loops allows for desirable mathematical properties of simple graphs, its absence is significant biologically. Network function is maintained by biologic feedback loops, which cannot be modeled with the current graph theory framework. These feedback loops could have particular importance in the distinction of fMRI connectivity patterns between controls and those with schizophrenia.

A future direction within this modeling approach would be to use the RPD and kernel logistic regression within a different, more confined brain atlas. The hybrid atlas contains 132 parcellated regions covering the entirely of the brain. However, it may be that restricting this methodology to pre-specified regions of interest may bear results more comparable to that seen in simulation. Additionally, as the RPD is scale invariant, relative, rather than absolute, differences in connectivity may be more informative for this algorithm. Specifically, if the total sample average connectivity between two nodes is some value *ρ* satisfying −1 < *ρ* < 1, then looking at differences in individual deviation values from this average, rather than the absolute differences, may help circumvent the scale invariance of the distance metric. Finally, extending this algorithm within a tensor machine context could overcome the issue of subtle differences within the functional conenctivity between groups.
